# Low‑Energy Fall With High‑Impact Consequences: A Case Report and Biomechanical Literature Review of Ogawa Type I Coracoid Process Avulsion Fracture With Acromioclavicular Joint Dislocation

**DOI:** 10.7759/cureus.107731

**Published:** 2026-04-26

**Authors:** Jhenica Uthayakumaran, Neil Ashwood, Shahmeen Rasul, Saud Danyowi

**Affiliations:** 1 Medicine and Surgery, Leicester Medical School, Leicester, GBR; 2 Trauma and Orthopaedics, University Hospitals of Derby and Burton National Health Service (NHS) Foundation Trust, Burton Upon Trent, GBR

**Keywords:** avulsion fracture, complex surgery, coracoid process, coracoid process fracture, orthopaedic surgery, shoulder injuries, upper limb surgery

## Abstract

Coracoid process fractures are rare injuries, usually associated with high-energy trauma and disruption of the superior shoulder suspensory complex (SSSC). We report the case of a 36-year-old male who sustained an Ogawa type I coracoid avulsion fracture with concomitant acromioclavicular (AC) joint dislocation following a low-energy mechanical fall. Initial radiographs demonstrated AC joint displacement with an associated coracoid fragment, further characterised by computed tomography (CT) with three-dimensional reconstruction. Imaging confirmed a displaced basal fracture involving the coracoclavicular ligament origin, representing a biomechanically unstable double disruption of the SSSC. Operative management consisted of open reduction and internal fixation of the coracoid with a cannulated lag screw combined with AC joint stabilisation using a hook plate and supplementary suture fixation. This case highlights the importance of recognising occult instability in uncommon shoulder injuries, the role of advanced imaging in operative planning, and the need to restore both bony and ligamentous continuity to re-establish shoulder girdle stability.

## Introduction

The coracoid process acts as a key stabilising structure within the shoulder girdle, serving as an attachment site for the coracoclavicular, coracoacromial, and coracohumeral ligaments, as well as the pectoralis minor, coracobrachialis, and short head of biceps muscles. Through these attachments, it plays an essential role in force transmission between the clavicle and scapula.

Coracoid fractures account for less than 1% of all fractures [1] and approximately 2-13% of scapular fractures [2], making them an uncommon injury encountered in clinical practice [3]. These injuries frequently coexist with disruption of the superior shoulder suspensory complex (SSSC), a ring-like stabilising structure described by Goss [4,5] comprising the glenoid, coracoid process, acromion, distal clavicle, and connecting ligaments. A single disruption may remain stable; however, a double disruption results in significant biomechanical instability and typically requires surgical stabilisation.

The Ogawa classification [6] categorises coracoid fractures according to their relationship to the coracoclavicular ligaments: (1) type I: proximal to the coracoclavicular ligaments [7] - unstable; (2) type II: distal (tip fractures) - generally stable; and (3) most type I injuries occur following high-energy trauma. This report describes an unusual low-energy mechanism producing a high-impact unstable injury pattern.

## Case presentation

A 36-year-old previously healthy male presented after a mechanical fall down a flight of stairs with direct impact to the right shoulder against a door. He reported immediate severe pain and inability to elevate the arm.

Clinical assessment revealed swelling and deformity over the AC joint with tenderness extending toward the coracoid process. Skin integrity was preserved. Distal neurovascular examination was normal.

**Figure 1:**
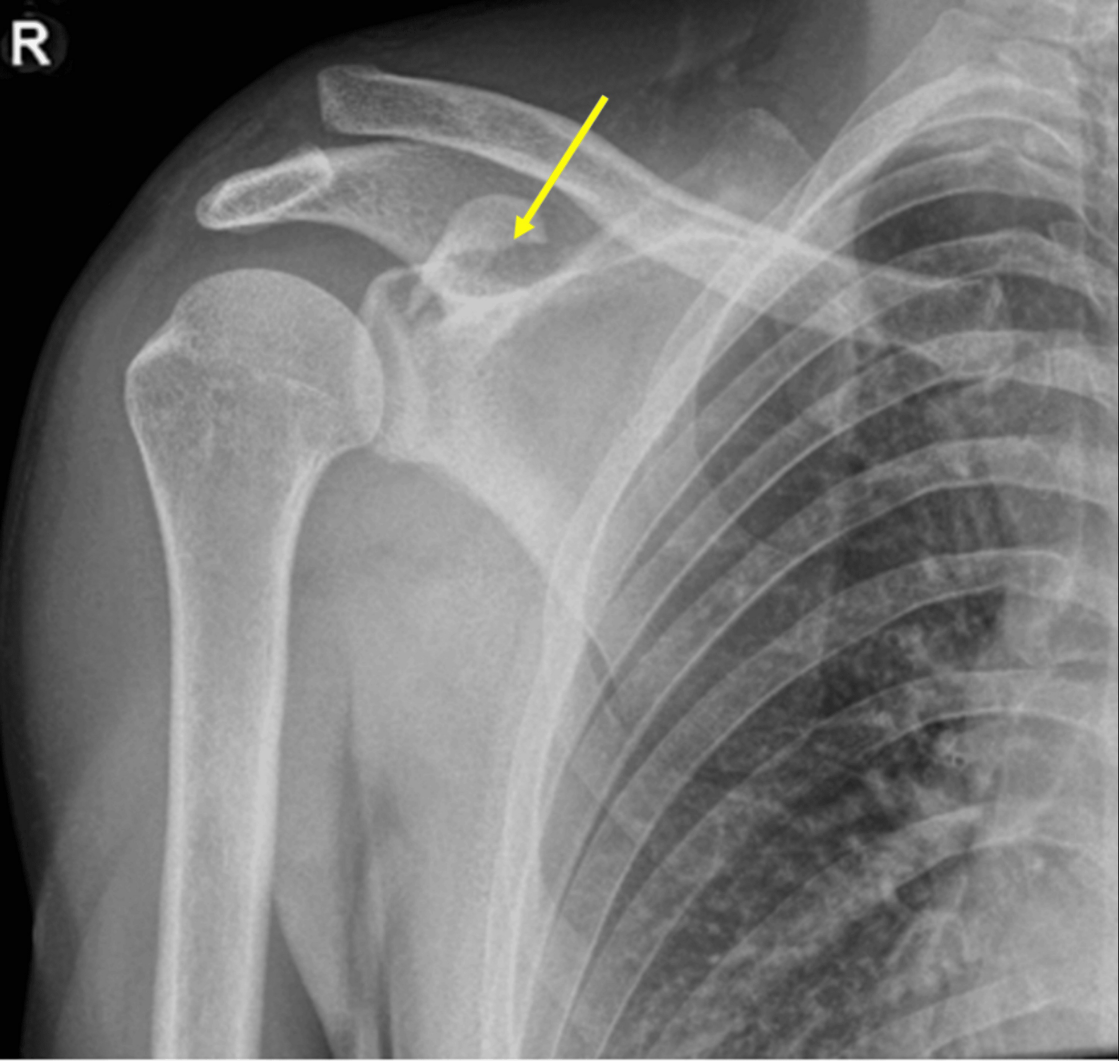
AP shoulder radiograph showing AC joint displacement with an associated coracoid fracture fragment The fractured coracoid process is indicated by the yellow arrow in this image. AC: acromioclavicular; AP: anterior-posterior

**Figure 2:**
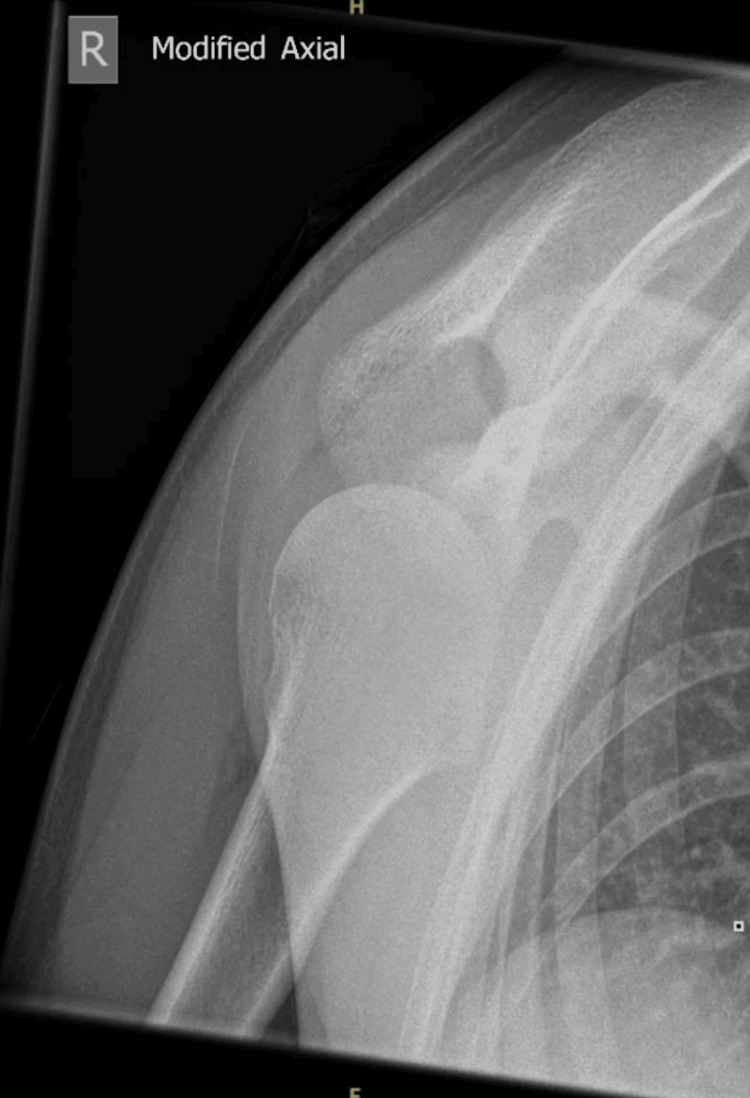
Lateral shoulder radiograph showing AC joint displacement with an associated coracoid fracture fragment AC: acromioclavicular

Plain radiographs (Figures 1-2) demonstrated superior displacement of the distal clavicle consistent with AC joint dislocation with an associated coracoid fracture fragment.

**Figure 3:**
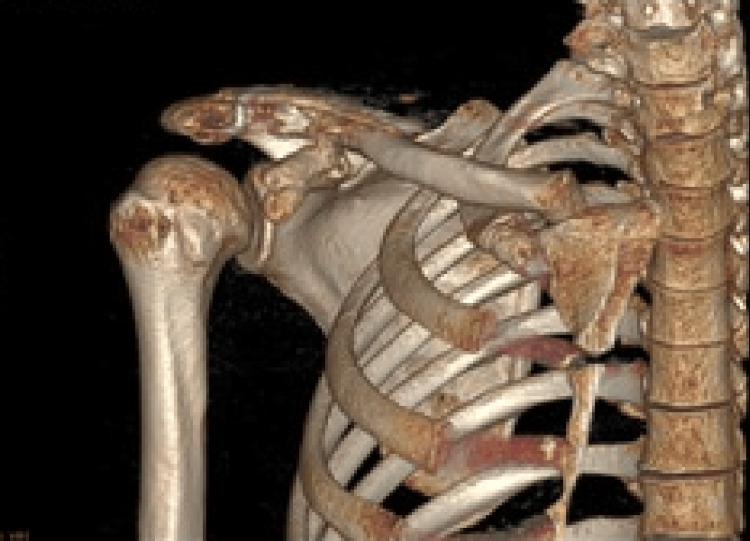
CT 3D reconstruction demonstrating a displaced Ogawa type I basal coracoid fracture

A computed tomography (CT) scan with 3D reconstruction was performed for preoperative planning (Figure 3). This confirmed a displaced fracture at the base of the coracoid process involving the origin of the coracoclavicular ligaments. CT imaging with three-dimensional reconstruction confirmed a displaced fracture at the base of the coracoid involving the origin of the coracoclavicular ligaments, consistent with an Ogawa type I fracture. The combined AC dislocation and coracoid fracture represented a double disruption of the SSSC.

Despite the apparently low-energy mechanism, the fracture pattern suggested an avulsion injury caused by tensile loading through the coracoclavicular ligaments during AC displacement rather than direct impact alone. Generally, scapular fractures, including the coracoid process, have been treated non-operatively with good outcomes. However, given the instability of the injury pattern and risk of persistent dysfunction with conservative treatment, operative management was undertaken.

Operative technique 

**Figure 4:**
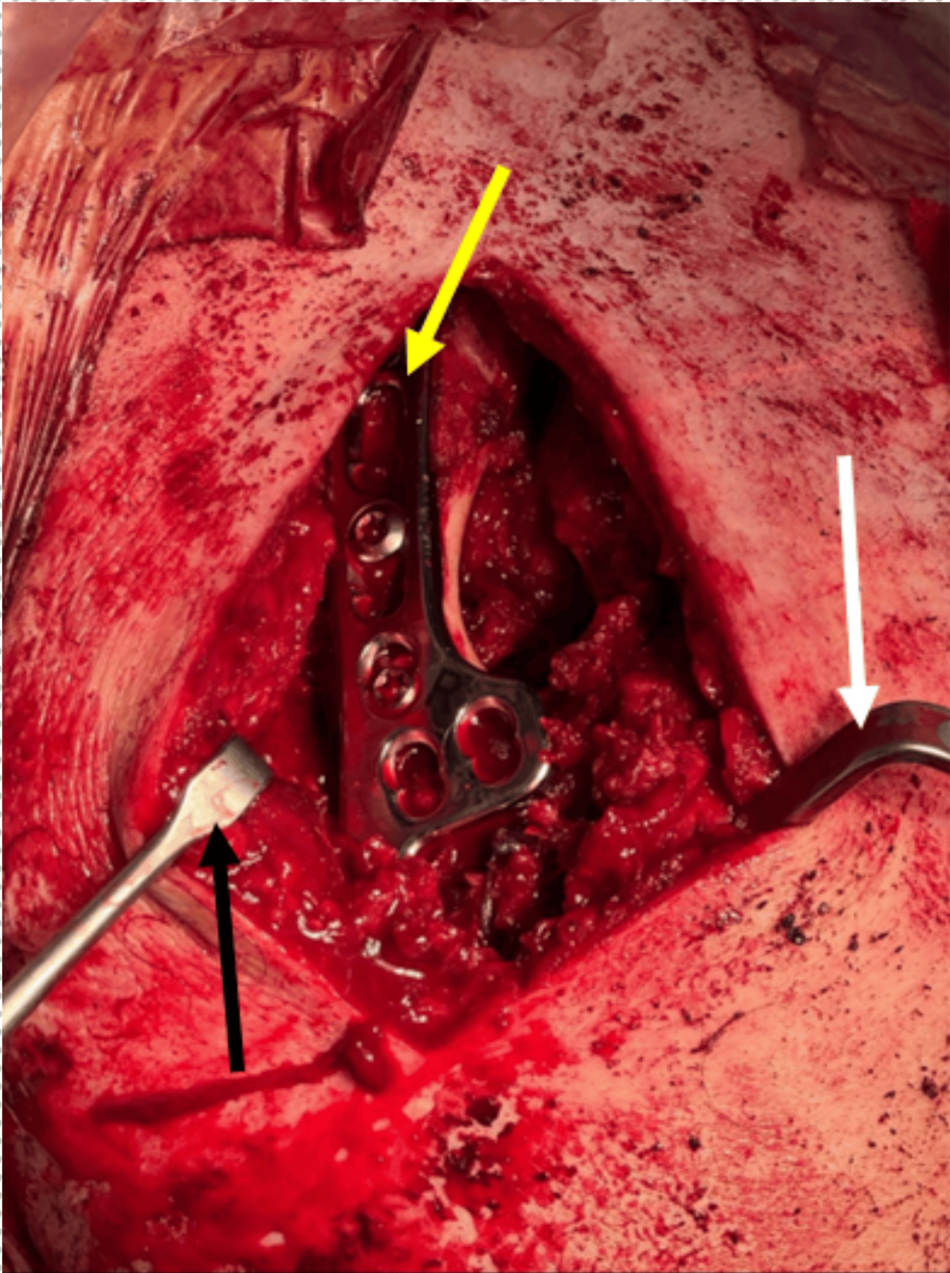
Intra-operative image showing hook plate AC stabilisation and screw fixation of the coracoid The black and white arrows demonstrate Langenbeck retractors used for adequate exposure. The yellow arrow indicates the hook plate in situ following fixation.

During surgery under anaesthesia, an anterior deltopectoral approach was utilised to expose the coracoid process, supplemented by a superior approach to the AC joint. A 1-cm segment of the distal clavicle was excised using an oscillating saw to facilitate reduction. Two 2.5-mm drill holes were created in the lateral clavicle and acromion, and No. 2 FiberTape was passed for provisional AC stabilisation. A 15-mm clavicular hook plate was applied to restore AC alignment and counteract superior displacement of the clavicle (Figure 4).

**Figure 5:**
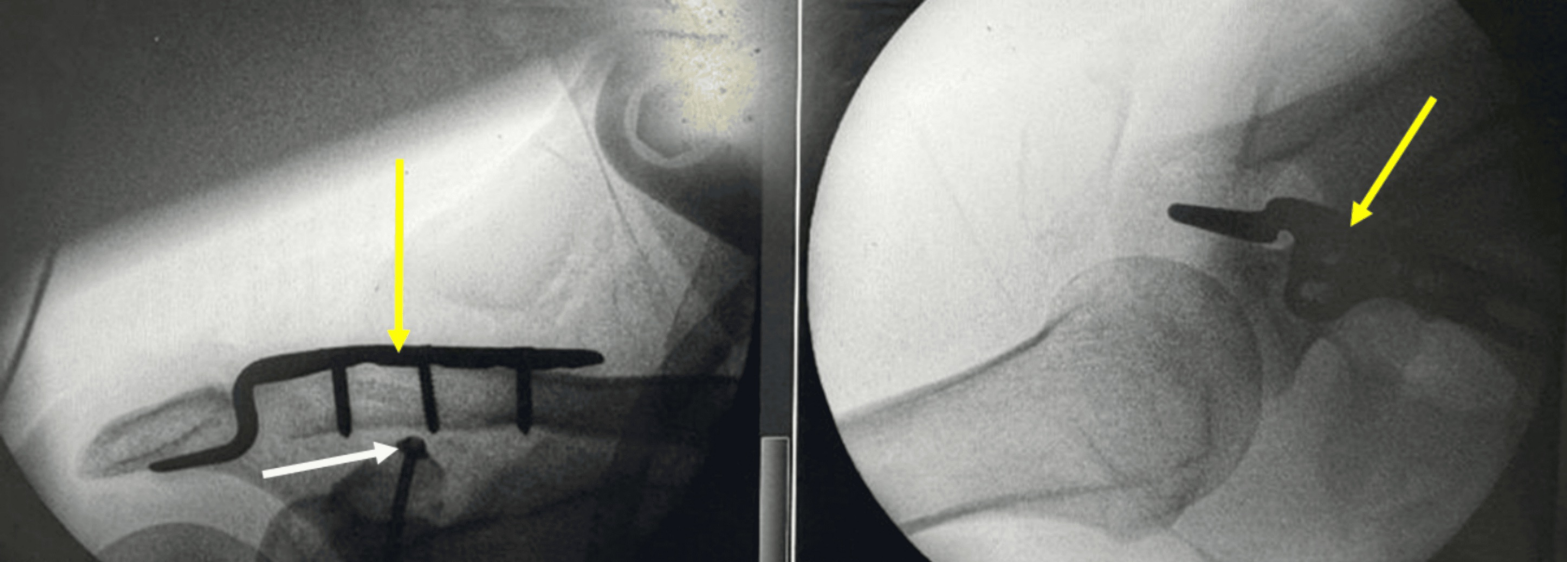
Intra-operative fluoroscopy showing successful reduction and fixation of a coracoid process fracture The white arrow highlights a cannulated lag screw, while the yellow arrows indicate the clavicular hook plate following completion of surgery.

The coracoid fragment was then anatomically reduced under direct visualisation and temporarily stabilised using a 1.6-mm Kirschner wire. Definitive fixation was achieved using a 4-mm cannulated lag screw with a washer, restoring compression and re-establishing attachment of the coracoclavicular ligaments. Intra-operative fluoroscopy (Figure 5) confirmed satisfactory reduction and fixation. 

Post-operative rehabilitation 

The shoulder was immobilised in a polysling for four weeks. Passive mobilisation commenced early, progressing to active movement following evidence of early healing. Resisted activity and lifting were restricted until radiographic union. Planned hook plate removal was scheduled at approximately three months to minimise subacromial irritation.

## Discussion

Coracoid fractures are rare and frequently underdiagnosed, particularly when associated with more obvious AC joint injuries [8]. The majority arise following high-energy trauma; therefore, the low-energy mechanism observed in this case represents an unusual presentation.

Biomechanical considerations 

Understanding this injury through the SSSC model [9] is essential. The AC dislocation constituted one disruption of the suspensory ring, while avulsion of the coracoid base created a second disruption. This eliminated the normal clavicle-scapula suspension system, resulting in vertical instability and altered load transfer across the shoulder girdle. 

Role of imaging 

Plain radiographs can underestimate injury severity due to overlapping anatomy and other injuries [10]. CT with three-dimensional reconstruction proved critical in identifying a basal type I fracture rather than a stable tip fracture, directly influencing treatment strategy [11].

Operative strategy 

Although stable type II coracoid fractures may be treated conservatively, type I fractures associated with SSSC disruption have been associated with displacement, pain, and functional limitation when treated non-operatively [12,13]. Fixation with a hook plate is an effective treatment for coracoid process fractures, as reported in literature [14], by restoring structural continuity and allowing predictable rehabilitation [15].

In this case, AC stabilisation was performed first to re-establish global alignment. Hook plate fixation resisted superior clavicular displacement, while screw fixation restored coracoid continuity and coracoclavicular function [16]. Alternative techniques, such as suture-button constructs, may be suitable when fragments are small [17]; however, screw fixation provides reliable interfragmentary compression when adequate bone stock exists.

Overall literature consensus (Table 1) suggests operative management for unstable type I fractures associated with SSSC disruption.

**Table 1:** Literature comparison AC: acromioclavicular; ORIF: open reduction and internal fixation; SSSC: superior shoulder suspensory complex

Study	Injury Pattern	Management	Outcome
Ogawa et al. [6]	Type I coracoid ± AC injury	ORIF	Good union & function
Eyres et al. [8]	Basal coracoid with SSSC injury	Surgical fixation	Restored stability
Habarta et al. [7]	Coracoid fractures (long-term series)	Mixed operative/non-operative	Better stability in operative type I cases
Present case	Type I + AC dislocation	Hook plate + lag screw	Stable reduction and recovery

## Conclusions

This case highlights that, due to their rarity, coracoid fractures are frequently overlooked on initial imaging. It illustrates the complexities of coracoid fractures and the need for advanced imaging, such as CT, to guide management by enabling accurate fracture classification and surgical planning. When an associated AC dislocation is present, clinicians should maintain a high index of suspicion for a double disruption of the SSSC. Despite a seemingly low-energy mechanism, the injury resulted in significant shoulder girdle instability for this patient. As Ogawa type I coracoid fractures are biomechanically unstable, effective management requires restoration of both the AC joint and coracoid integrity to re-establish stability of the shoulder girdle. Early recognition, advanced imaging, and restoration of both bony and ligamentous continuity are what we have identified from our case as critical for achieving stable fixation and favourable functional recovery.

## References

[1] (2026). Scapula fractures. https://www.orthobullets.com/trauma/1013/scapula-fractures.

[2] Morgan M, Walizai T, Elfeky M (2026). Coracoid process fracture. Radiopaedia.org.

[3] Ada JR, Miller ME (1991). Scapular fractures: analysis of 113 cases. Clin Orthop Relat Res.

[4] Goss TP (1993). Double disruptions of the superior shoulder suspensory complex. J Orthop Trauma.

[5] Goss TP (1995). Scapular fractures and dislocations: diagnosis and treatment. J Am Acad Orthop Surg.

[6] Ogawa K, Yoshida A, Takahashi M, Ui M (1997). Fractures of the coracoid process. J Bone Joint Surg Br.

[7] Habarta J, Färber C, Jordan M, Gilbert F, Meffert R, Schmalzl J (2025). Coracoid fractures: long-term results and modification of the classification. Eur J Orthop Surg Traumatol.

[8] Eyres KS, Brooks A, Stanley D (1995). Fractures of the coracoid process. J Bone Joint Surg Br.

[9] Ogawa K, Matsumura N, Ikegami H (2012). Coracoid fractures: therapeutic strategy and surgical outcomes. J Trauma Acute Care Surg.

[10] Galvin JW, Kang J, Ma R, Li X (2020). Fractures of the coracoid process: evaluation, management, and outcomes. J Am Acad Orthop Surg.

[11] (2026). ORIF - lag screw fixation: body and processes, coracoid. https://surgeryreference.aofoundation.org/orthopedic-trauma/adult-trauma/scapula/body-and-processes-coracoid/orif-lag-screw-fixation#approach.

[12] Anavian J, Wijdicks CA, Schroder LK, Vang S, Cole PA (2009). Surgery for scapula process fractures: good outcome in 26 patients. Acta Orthop.

[13] Kuhn JE, Blasier RB, Carpenter JE (1994). Fractures of the acromion process: a proposed classification system. J Orthop Trauma.

[14] Ye CX, Guo YB, Zheng YH, Wu ZB, Chen KY, Zhang XL, Chen ZM (2023). Treatment of coracoid process fractures combined with acromioclavicular joint dislocation using clavicular hook plate. J Shoulder Elbow Surg.

[15] Li X, Ma R, Bedi A, Dines DM, Altchek DW, Dines JS (2014). Management of acromioclavicular joint injuries. J Bone Joint Surg Am.

[16] Kennedy JC, Cameron H (1954). Complete dislocation of the acromio-clavicular joint. J Bone Joint Surg Br.

[17] Dunn JC, Waterman BR (2015). Successful nonoperative management of coracoid fracture associated with suture-button fixation of acromioclavicular separation. Mil Med.

